# Optimizing Perioperative Management of Pancreatic Ductal Adenocarcinoma: Insights Into Modified FOLFIRINOX Relative Dose Intensity and CA 19‐9 Dynamics

**DOI:** 10.1002/jso.70057

**Published:** 2025-07-31

**Authors:** Jiage Qian, Nikhil V. Tirukkovalur, Janie Y. Zhang, Anwaar Saeed, Sebastiaan Ceuppens, Robin Schmitz, Aatur Singhi, Kenneth K. Lee, Amer H. Zureikat, Alessandro Paniccia

**Affiliations:** ^1^ University of Pittsburgh School of Medicine Pittsburgh Pennsylvania USA; ^2^ Division of Surgical Oncology University of Pittsburgh Medical Center Pittsburgh Pennsylvania USA; ^3^ Department of Medical Oncology University of Pittsburgh Medical Center Pittsburgh Pennsylvania USA; ^4^ Department of Pathology University of Pittsburgh Medical Center Pittsburgh Pennsylvania USA

**Keywords:** CA 19‐9 normalization, modified FOLFIRINOX, pancreatic ductal adenocarcinoma, relative dose intensity, survival

## Abstract

**Background:**

Many patients with resectable pancreatic ductal adenocarcinoma (PDAC) treated with modified FOLFIRINOX (mFOLFIRINOX) require dose reduction due to adverse effects. This study explores the optimal threshold for mFOLFIRINOX relative dose intensity (RDI) and characterizes RDI's correlation with CA 19‐9.

**Methods:**

A single‐institution retrospective analysis of 97 patients with PDAC treated with mFOLFIRINOX and pancreatectomy from 2017 to 2022. RDI was calculated by dividing the delivered dose intensity by the intended dose intensity over 6 months.

**Results:**

Median overall RDI was 73.8% (fluorouracil 75.5%, irinotecan 74.5%, oxaliplatin 70.6%). An RDI cutoff of ≥ 70% (*n* = 57) was associated with significantly improved overall survival (median OS: 62.6 vs. 43.7 months, *p* = 0.034). Compared to patients with < 70% RDI who did not achieve CA 19‐9 normalization, those with ≥ 70% RDI and normalization had significantly improved survival (HR: 0.27; 95% CI: 0.11–0.73). No significant benefit was observed with ≥ 70% RDI without CA 19‐9 normalization or < 70% RDI with normalization. In the multivariable model, RDI ≥ 70% remained independently associated with improved OS (HR = 0.37, 95% CI: 0.18–0.79) but not disease‐free survival (HR = 0.50, 95% CI: 0.24–1.03).

**Conclusion:**

Receiving ≥ 70% RDI of mFOLFIRINOX and CA 19‐9 normalization independently improves survival in resected PDAC. The greatest benefit is observed when both are achieved.

## 1 Introduction

Modified FOLFIRINOX (mFOLFIRINOX) is an established initial treatment for patients diagnosed with pancreatic ductal adenocarcinoma (PDAC) [1, 2, 3]. However, there is still active debate regarding the optimal treatment sequencing. Early randomized trials demonstrated that surgical resection followed by adjuvant chemotherapy with mFOLFIRINOX improved disease‐free survival (DFS) and overall survival (OS). In practice, however, many patients fail to complete adjuvant therapy [1, 4], with reports indicating that only 35%–81% of patients initiate adjuvant therapy, and as few as 7%–65% complete the intended course [5, 6, 7, 8]. These challenges have driven interest in neoadjuvant FOLFIRINOX, which improves systemic therapy delivery and demonstrates improved R0‐resection rates and median OS [3, 9, 10, 11].

A parallel yet understudied aspect of optimizing treatment sequencing is a refined measurement of chemotherapy delivery. Traditional cycle‐based metrics are insufficient, as they do not reflect dose modifications due to toxicity. Relative dose intensity (RDI), which accounts for both the quantity and rate of chemotherapy administered, has been proposed as a more precise metric [12]. Studies have demonstrated that higher RDI (with cut‐offs ranging from 56% to 80%) correlates with improved survival in resected PDAC [13, 14, 15]. Despite these new findings, critical gaps remain. Specifically, the distribution of RDI across neoadjuvant and adjuvant settings, its concordance with traditional cycle‐based calculations, and its relationship with CA 19‐9 dynamics remain poorly understood.

Building on previous findings, this study evaluates the association between perioperative RDI and chemotherapy cycles, CA 19‐9 normalization, and survival outcomes. By examining these nuances, this study aims to define an RDI cutoff that correlates with survival and examine its clinical implications in patients who underwent surgery for PDAC.

## 2 Methods

### 2.1 Cohort Selection

**Figure 1:**
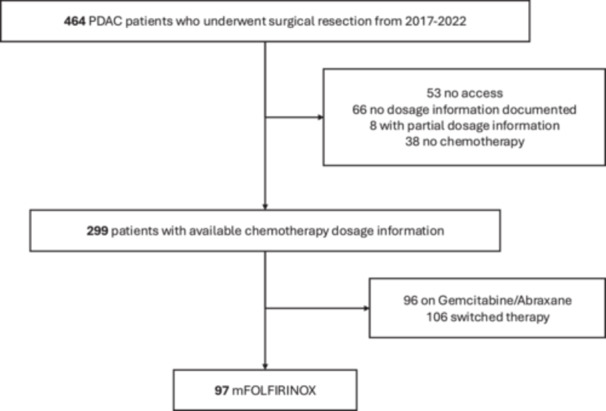
Study cohort selection flowchart. mFOLFIRINOX, modified FOLFIRINOX; PDAC, pancreatic ductal adenocarcinoma.

This study retrospectively reviewed patients with PDAC who underwent pancreatectomy between 2017 and 2022 at a single institution (Figure 1). Patients were selected if they received single‐regimen mFOLFIRINOX therapy in the perioperative setting and had available chemotherapy dosage data. The final cohort included 97 patients.

### 2.2 RDI Calculation

Supporting Information S1: Figure S1 presents the calculation of single‐agent RDI utilized in this study. The intended therapy dosage was based on a 6‐month (24‐week) treatment course, with specific intended dose intensities per cycle: fluorouracil (5FU; 2400 mg/m²), irinotecan (150 mg/m²), and oxaliplatin (85 mg/m²), administered over 46 h every 2 weeks. This corresponded to a total 6‐month dosage of 5FU 28 800 mg/m², irinotecan 1800 mg/m², and oxaliplatin 1020 mg/m² (c in Supporting Information S1: Figure S1).

To calculate the RDI, the total dose received (a) was divided by the therapy duration in weeks to determine the unadjusted RDI (d). This value was then multiplied by the adjustment factor (e), which was the ratio of the total dose received (a) to the total intended dose (c). The adjustment factor accounted for any deviations from the intended dose. The final adjusted RDI (f) was obtained by multiplying the unadjusted RDI (d) by the adjustment factor (e). Multiagent RDI was determined by averaging the three single‐agent RDIs.

### 2.3 Statistical Analysis

Demographic, preoperative, and postoperative characteristics according to RDI level were analyzed using the Wilcoxon rank‐sum and Pearson *χ* ^2^ tests, as appropriate. Kaplan–Meier survival curves and univariable analyses using the log‐rank test were used to evaluate OS and DFS in relation to RDI, both independently and in interaction with normalization of CA 19‐9 at the end of therapy (defined as after surgery or after adjuvant therapy, if administered). A multivariable Cox proportional hazards model was used to investigate the independent effect of RDI on OS and DFS. Finally, logistic regression analysis was performed to identify predictors of receiving a high RDI. Statistical analyses were conducted using Stata (version 17.0, StataCorp LLC, College Station, TX).

## 3 Results

The median overall RDI was 73.8%, with single‐agent RDIs being 75.5%, 74.5%, and 70.6% for 5FU, irinotecan, and oxaliplatin, respectively. In the neoadjuvant setting, the median RDI was 52.8% (5FU: 51.3%; Irinotecan: 55.9%; Oxaliplatin: 46.2%).

### 3.1 Clinicopathological Characteristics

The median age of the cohort was 64.3 years (Table 1; IQR: 59.1–71.6), with 57.7% of patients being male and a median BMI of 26.3 (IQR: 23.1–29.9). A RDI cutoff of 70% was informed by the cohort's median RDI (73.8%) and supported by restricted cubic spline analysis (Supporting Information S2: Figure S2), which demonstrated a continuous decline in hazard beyond this point. No significant differences were observed between patients receiving < 70% RDI and those receiving ≥ 70% RDI in terms of age, sex, BMI, history of diabetes, hypertension, or median CA19‐9 at diagnosis. Most patients presented with stage 1 (37.5% in < 70% RDI vs. 29.8% in ≥ 70% RDI) and stage 2 (32.5% in < 70% RDI vs. 43.9% in ≥ 70% RDI) disease, with no significant differences in clinical stage distribution (*p *= 0.40). Of note, patients with ≥ 70% total RDI had a median neoadjuvant RDI of 58.7%, compared to 40.6% in those with < 70% total RDI (*p *< 0.001; Table 1).

**Table 1:** Clinicopathologic characteristics based on relative dose intensity.

	Total	< 70% RDI	≥ 70% RDI	*p* value
Characteristics	(*n* = 97)	(*n* = 40)	(*n* = 57)	
Demographics				
Age	64.3 (59.1–71.6)	63.5 (58.3–73.5)	64.8 (60.7–69.0)	0.83
Sex (male)	57.7%	47.5%	64.9%	0.087
BMI	26.3 (23.1–29.9)	26.7 (24.3–30.1)	25.6 (22.7–29.3)	0.68
History of diabetes	27.8%	25.0%	29.8%	0.60
History of hypertension	47.4%	42.5%	50.9%	0.42
History of cardiovascular disease	8.2%	7.5%	8.8%	0.82
Other cancer history	22.7%	30.0%	17.5%	0.15
History of PE/DVT	4.1%	5.0%	3.5%	0.72
Preoperative characteristics				
Clinical stage				0.40
Stage 1	33.0%	37.5%	29.8%	
Stage 2	39.2%	32.5%	43.9%	
Stage 3	1.0%	0.0%	1.8%	
Missing	26.8%	30.0%	24.6%	
CA 19‐9 at diagnosis	92.7 (18.0–437.7)	50.0 (9.1–328.0)	115.0 (44.5–547.1)	0.67
Preoperative CA 19‐9	30.0 (13.5–69.1)	30.0 (8.0–76.0)	29.0 (16.0–63.0)	0.42
CA 19‐9 secretors (≥ 37 U/mL)	67.0%	52.5%	77.2%	0.011
Preoperative CA 19‐9 normalization among secretors	43.1%	28.6%	50.0%	0.10
Neoadjuvant RDI	52.8%	40.6%	58.7%	< 0.001
Operative variables				
Procedure				0.50
Whipple	75.3%	70.0%	78.9%	
Distal pancreatectomy	21.6%	27.5%	17.5%	
Total pancreatectomy	3.1%	2.5%	3.5%	
Approach				0.49
Open	55.7%	55.0%	56.1%	
Robotic/laparoscopic	44.3%	45.0%	43.9%	
Gland texture				0.44
Soft	12.4%	15.0%	10.5%	
Hard	59.8%	55.0%	63.2%	
Unknown	27.8%	30.0%	26.3%	
Pathological features				
Pathologic T‐stage				0.48
T1	46.4%	42.5%	49.1%	
T2	43.3%	50.0%	38.6%	
T3	10.3%	7.5%	12.3%	
Pathologic N‐stage				0.66
N0	42.3%	37.5%	45.6%	
N1	32.0%	32.5%	31.6%	
N2	25.8%	30.0%	22.8%	
Positive lymph node ratio				0.60
0%	43.3%	37.5%	47.4%	
1%–20%	35.1%	40.0%	31.6%	
> 20%	21.6%	22.5%	21.1%	
Margin				0.15
R0	68.0%	60.0%	73.7%	
R1	32.0%	40.0%	26.3%	
Lymphovascular invasion	71.9%	80.0%	66.1%	0.13
Perineural invasion	85.6%	95.0%	78.9%	0.03
Postoperative complications				
Delayed gastric emptying	16.5%	15.0%	17.5%	0.74
Any POPF	12.4%	22.5%	5.3%	0.01
CR‐POPF (grade B/C)	7.2%	15.0%	1.8%	0.01
Fluid collection	16.5%	20.0%	14.0%	0.44
Postoperative hemorrhage	2.1%	5.0%	0.0%	0.09
Total length of stay	6 (5–8)	7 (5–10)	6 (5–7)	0.81
Reoperation	8.2%	5.0%	10.5%	0.33
Readmission	26.8%	42.5%	15.8%	0.003

*Note:* Continuous variables are presented as median (interquartile range). Other cancer history encompasses any cancer, including skin cancer.

Abbreviations: CR‐POPF, clinically relevant postoperative pancreatic fistula; POPF, postoperative pancreatic fistula; RDI, relative dose intensity.

Perineural invasion was more common in the < 70% RDI group (95.0% vs. 78.9%, *p* = 0.027). Patients receiving < 70% RDI also had higher rates of any postoperative pancreatic fistula (POPF; 22.5% vs. 5.3%, *p* = 0.011), clinically relevant POPF (CR‐POPF; 15.0% vs. 1.8%, *p* = 0.013), and readmissions (42.5% vs. 15.8%, *p* = 0.003). No significant differences were observed with respect to delayed gastric emptying, fluid collection, postoperative hemorrhage, length of stay, or reoperation rates.

### 3.2 Correlation With Chemotherapy Cycles

Three time points (2, 4, and 6 months of therapy) were selected to assess the relationship between RDI and the number of chemotherapy cycles (Supporting Information S1: Figure S3). At 2 months (4 cycles), the median RDI was 25.0% (IQR: 18.2%–35.5%). By 4 months (8 cycles), the median RDI increased to 61.7% (IQR: 54.2%–76.5%), and at 6 months (12 cycles), the median RDI further increased to 92.6% (IQR: 76.8%–100.2%).

### 3.3 Overall and Disease‐Free Survival

**Figure 2:**
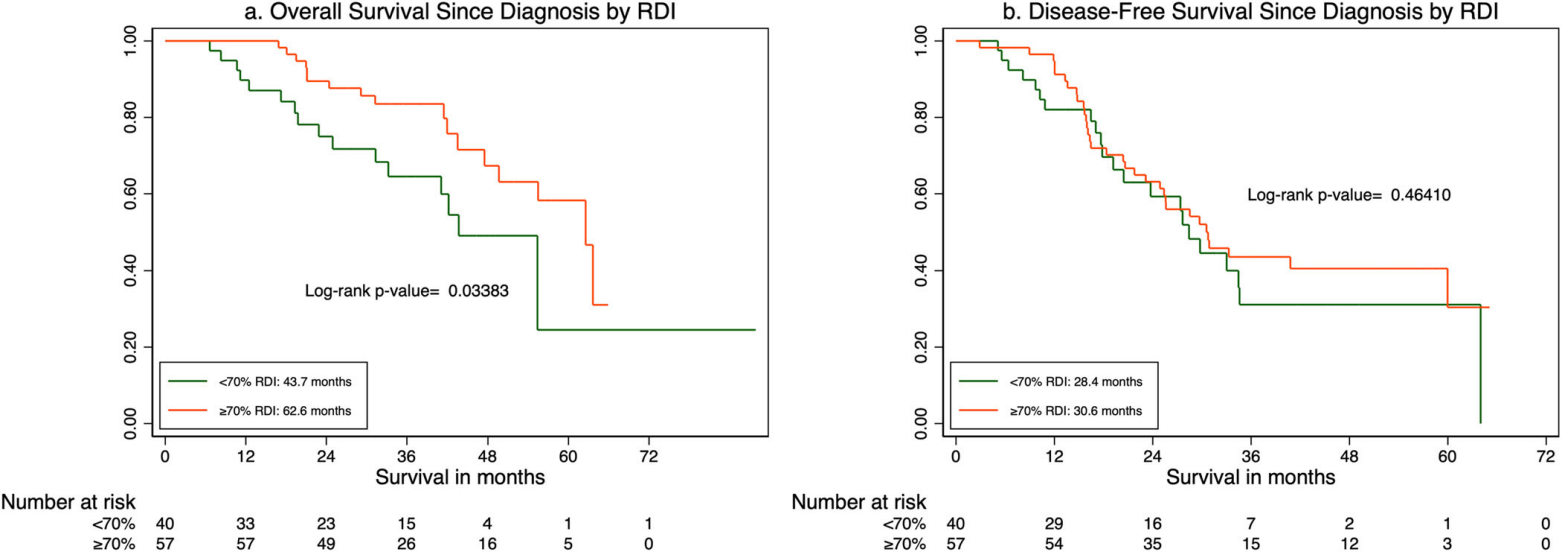
Overall and disease‐free survival by relative dose intensity. RDI, relative dose intensity. (a) Overall survival since diagnosis by RDI. (b) Disease‐free survival since diagnosis by RDI.

The median OS for patients with an RDI ≥ 70% was 62.6 months, compared to 43.7 months in those with an RDI < 70% (Figure 2a; log‐rank *p*= 0.034). The median DFS was 30.6 months in the ≥ 70% RDI group versus 28.4 months in the < 70% RDI group, although this difference did not reach statistical significance (Figure 2b; log‐rank *p* = 0.46).

As shown in Table 2, ≥ 70% RDI was associated with a HR of 0.48 in OS (95% CI: 0.24–0.96). For DFS, diagnostic CA 19‐9 > 500 U/mL (HR: 2.04, 95% CI: 1.11–3.75, *p* = 0.023), T2/T3‐stage (HR: 2.32, 95% CI: 1.27–4.25, *p* = 0.007; HR: 4.18, 95% CI: 1.78–9.84, *p* = 0.001), N2‐stage (HR: 4.03, 95% CI: 2.11–7.69, *p* < 0.001), positive lymph node ratio > 20% (HR: 3.88, 95% CI: 1.98–7.60, *p* < 0.001), R1 margin (HR: 2.53, 95% CI: 1.27–5.04, *p* = 0.009), and perineural invasion (HR: 3.08, 95% CI: 1.11–8.57, *p* = 0.031) were associated with increased disease progression. In contrast, CA 19‐9 normalization at the end of total therapy (i.e., neoadjuvant, surgery, and adjuvant, if administered) was linked to a lower progression risk (HR: 0.52, 95% CI: 0.28–0.95, *p* = 0.035).

**Table 2:** Univariable Cox regression on overall and disease‐free survival.

	Overall survival		Disease‐free survival	
	HR (95% CI)	*p* value	HR (95% CI)	*p* value
RDI ≥ 70%	0.48 (0.24, 0.96)	0.038	0.60 (0.30, 1.18)	0.135
Age (ref: < 65)	1.04 (0.52, 2.07)	0.761	1.14 (0.67, 1.93)	0.639
Sex (ref: female)	1.44 (0.67, 3.06)	0.355	1.15 (0.67, 2.00)	0.625
BMI	1.03 (0.97, 1.10)	0.386	1.03 (0.98, 1.08)	0.395
Comorbidity (ref: no)	0.82 (0.29, 2.35)	0.707	1.56 (0.56, 4.37)	0.404
CA 19‐9 at diagnosis (ref: 37–500)				
< 37	0.40 (0.15, 1.06)	0.064	0.60 (0.30, 1.20)	0.143
> 500	1.10 (0.50, 2.40)	0.830	2.04 (1.11, 3.75)	0.023
Preoperative CA 19‐9 normalization among secretors	0.81 (0.38, 1.72)	0.586	0.92 (0.50, 1.69)	0.794
End of therapy CA 19‐9 normalization among secretors	0.52 (0.25, 1.10)	0.088	0.52 (0.28, 0.95)	0.035
RDI and CA 19‐9 normalization interaction (ref: < 70%; not normalized)				
≥ 70%; not normalized	0.41 (0.14, 1.18)	0.098	0.86 (0.35, 2.14)	0.747
< 70%; normalized	0.56 (0.15, 2.10)	0.387	0.70 (0.25, 1.98)	0.504
≥ 70%; normalized	0.28 (0.11, 0.73)	0.009	0.43 (0.19, 0.97)	0.042
Procedure (ref: Whipple)				
Distal pancreatectomy	1.11 (0.48, 2.57)	0.823	1.08 (0.47, 2.51)	0.863
Total pancreatectomy	0.99 (0.14, 7.36)	0.992	1.38 (0.19, 10.21)	0.758
Approach (ref: open)				
Robotic/laparoscopic	0.73 (0.35, 1.49)	0.374	0.61 (0.35, 1.06)	0.074
Pathologic T‐stage (ref: T1)				
T2	1.60 (0.76, 3.38)	0.225	2.32 (1.27, 4.25)	0.007
T3	1.68 (0.48, 5.96)	0.422	4.18 (1.78, 9.84)	0.001
Pathologic N‐stage (ref: N0)				
N1	1.16 (0.48, 2.81)	0.561	1.52 (0.77, 3.00)	0.237
N2	2.07 (0.90, 4.77)	0.059	4.03 (2.11, 7.69)	< 0.001
Positive lymph node ratio (ref: 0%)				
1%–20%	1.54 (0.69, 3.43)	0.299	1.87 (0.98, 3.57)	0.060
> 20%	2.07 (0.86, 5.01)	0.108	3.88 (1.98, 7.60)	< 0.001
Margin (ref: R0)				
R1	2.02 (0.99, 4.09)	0.054	2.53 (1.27, 5.04)	0.009
Lymphovascular invasion (ref: no)	1.77 (0.77, 4.09)	0.186	1.81 (0.95, 3.44)	0.073
Perineural invasion (ref: no)	1.60 (0.56, 4.62)	0.385	3.08 (1.11, 8.57)	0.031
CR‐POPF (grade B/C; ref: no)	0.94 (0.23, 3.92)	0.925	0.57 (0.18, 1.85)	0.345
Reoperation (ref: no)	0.57 (0.14, 2.40)	0.442	1.27 (0.55, 2.96)	0.589
Readmission (ref: no)	1.21 (0.58, 2.53)	0.614	1.05 (0.57, 1.93)	0.891

Abbreviations: CI, confidence interval; CR‐POPF, clinically relevant postoperative pancreatic fistula; HR, hazard ratio; RDI, relative dose intensity.

In the multivariable analysis, RDI ≥ 70% remained independently associated with improved OS (Table 3; HR = 0.38; 95% CI: 0.18–0.79; *p* = 0.015), as did CA 19‐9 < 37 at diagnosis (HR = 0.27; 95% CI: 0.09–0.81; *p* = 0.019). For DFS, diagnostic CA 19‐9 > 500 (HR = 2.02; 95% CI: 1.07–3.84; *p* = 0.033) and N2‐stage (HR = 2.21; 95% CI: 1.12–4.02; *p* = 0.047) were associated with higher recurrence/metastases risk, while CA 19‐9 < 37 at diagnosis trended toward improved DFS (HR = 0.48; 95% CI: 0.23–1.01; *p* = 0.056).

**Table 3:** Multivariable Cox regression on overall and disease‐free survival.

	Overall survival		Disease‐free survival	
	aHR (95% CI)	*p* value	aHR (95% CI)	*p* value
RDI ≥ 70% (ref: < 70%)	0.38 (0.18, 0.79)	0.015	0.76 (0.42, 1.36)	0.387
≥ 70%				
CA 19‐9 at diagnosis (ref: 37–500)				
< 37	0.27 (0.09, 0.81)	0.019	0.48 (0.23, 1.01)	0.056
> 500	0.96 (0.38, 2.37)	0.924	2.02 (1.07, 3.84)	0.033
Pathological N stage (ref: N0)				
N1	1.02 (0.41, 2.55)	0.907	1.45 (0.73, 2.88)	0.295
N2	1.67 (0.68, 4.03)	0.262	2.21 (1.12, 4.02)	0.047
Pathological T stage (ref: T1)				
T2	1.05 (0.41, 2.65)	0.923	1.30 (0.65, 2.60)	0.456
T3	0.88 (0.19, 3.98)	0.868	2.06 (0.98, 5.02)	0.153
Margin (ref: R0)				
R1	1.64 (0.78, 3.38)	0.193	1.69 (0.93, 3.05)	0.083
Perineural invasion	1.12 (0.33, 2.84)	0.855	2.36 (0.75, 7.49)	0.143

Abbreviations: aHR, adjusted hazard ratio; CI, confidence interval; RDI, relative dose intensity.

### 3.4 Interplay Between RDI and CA 19‐9 Normalization

**Figure 3:**
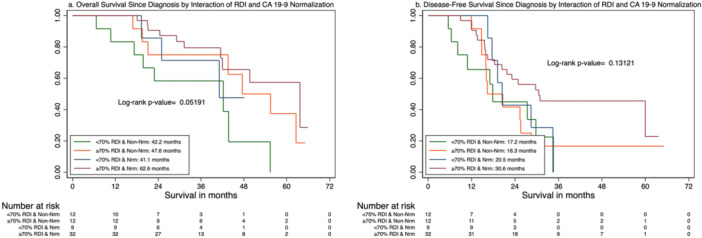
Overall and disease‐free survival by interaction between RDI and CA 19‐9 normalization. Nrm, CA 19‐9 normalization; Non‐Nrm, non‐normalization; RDI, relative dose intensity. (a) Overall survival since diagnosis by interaction of RDI and CA 19‐9 normalization. (b) Disease‐free survival since diagnosis by interaction of RDI and CA 19‐9 normalization.

Among patients with CA 19‐9 > 37 at diagnosis, completing ≥ 70% RDI was associated with increased odds of CA 19‐9 normalization at the end of total therapy (OR = 3.56, 95% CI: 1.20–10.57, *p*= 0.022). In the neoadjuvant setting, a cutoff of 44% was found to be associated with a higher likelihood of achieving preoperative normalization (OR = 3.67, 95% CI: 1.00–13.42, *p*= 0.050). When an interaction term was introduced to assess the combined effect of CA 19‐9 normalization at the end of therapy, the greatest survival benefit was observed in patients who achieved both ≥ 70% RDI and CA 19‐9 normalization (Table 2 and Figure 3; HR for OS: 0.28, 95% CI: 0.11–0.73, *p* = 0.009; HR for DFS: 0.43, 95% CI: 0.19–0.97, *p* = 0.042), whereas those achieving only one of these factors showed less pronounced, nonsignificant improvements (≥ 70% RDI without normalization: HR for OS: 0.41, 95% CI: 0.14–1.18, *p* = 0.098; HR for DFS: 0.86, 95% CI: 0.35–2.14, *p* = 0.74; < 70% RDI with normalization: HR for OS: 0.56, 95% CI: 0.15–2.10, *p* = 0.39; HR for DFS: 0.70, 95% CI: 0.25–1.98, *p* = 0.150).

### 3.5 Predictors of Achieving ≥ 70% RDI

As shown in Table 4, receiving ≥ 44% neoadjuvant RDI significantly increased the likelihood of achieving ≥ 70% total RDI (OR = 6.95, 95% CI: 2.58–18.71, *p* < 0.001). In contrast, postoperative complications were negative predictors, with readmission (OR = 0.26, 95% CI: 0.10–0.66, *p* = 0.005) and CR‐POPF (OR = 0.11, 95% CI: 0.02–0.88, *p* = 0.038) significantly reducing the odds.

**Table 4:** Predictors of receiving ≥ 70% RDI.

	OR (95% CI)	*p* value
Neoadjuvant RDI ≥ 44% (ref: < 44%)	6.95 (2.58, 18.71)	< 0.001
Age (ref: < 65)	1.31 (0.58, 2.95)	0.520
Sex (ref: female)	2.05 (0.90, 4.67)	0.089
BMI	0.99 (0.92, 1.06)	0.672
Comorbidity (ref: no)	0.38 (0.08, 1.92)	0.239
Clinical stage (ref: stage 1)		
Stage 2	0.88 (0.34, 2.28)	0.787
Stage 3	0.66 (0.24, 1.79)	0.404
CA 19‐9 at diagnosis (ref: 37‐500)		
< 37	0.35 (0.14–0.92)	0.034
> 500	1.26 (0.42–3.75)	0.679
Preoperative CA 19‐9 normalization among secretors (ref: not normalized)	2.50 (0.82, 7.64)	0.108
Procedure (ref: Whipple)		
Distal pancreatectomy	0.57 (0.22, 1.51)	0.253
Total pancreatectomy	1.25 (0.11, 14.37)	0.861
Approach (ref: open)		
Robotic/laparoscopic	1.02 (0.45, 2.30)	0.979
Gland texture (ref: soft)		
Hard	1.64 (0.47, 5.71)	0.440
Pathologic T‐stage (ref: T1)		
T2	0.67 (0.29, 1.57)	0.354
T3	1.42 (0.33, 6.23)	0.645
Tumor size (ref: < 2 cm)		
2.1–4 cm	0.70 (0.30, 1.64)	0.401
> 4 cm	1.68 (0.40, 7.23)	0.486
Pathologic N‐stage (ref: N0)		
N1	0.80 (0.31, 2.08)	0.645
N2	0.63 (0.23, 1.72)	0.362
Positive lymph node ratio (ref: 0%)		
1%–20%	0.63 (0.25, 1.58)	0.318
> 20%	0.75 (0.26, 2.16)	0.583
Margin (ref: R0)		
R1	0.54 (0.23, 1.28)	0.157
Lymphovascular invasion (ref: no)	0.49 (0.19, 1.27)	0.138
Perineural invasion (ref: no)	0.20 (0.05, 0.94)	0.041
Delayed gastric emptying (ref: no)	1.21 (0.40, 3.64)	0.740
CR‐POPF (grade B/C; ref: no)	0.11 (0.02, 0.88)	0.038
Fluid collection (ref: no)	0.66 (0.23, 1.92)	0.438
Total length of stay	0.99 (0.91, 1.08)	0.810
Reoperation (ref: no)	0.26 (0.10, 0.66)	0.005
Readmission (ref: no)	2.24 (0.43, 11.7)	0.341

Abbreviations: CI, confidence interval; CR‐POPF, clinically relevant postoperative pancreatic fistula; OR, odds ratio; POPF, postoperative pancreatic fistula; RDI, relative dose intensity.

## 4 Discussion

This study provides a clinically relevant threshold for RDI in PDAC, demonstrating that achieving ≥ 70% mFOLFIRINOX RDI significantly improves OS, particularly when combined with CA 19‐9 normalization. Furthermore, we identified ≥ 44% neoadjuvant RDI as a strong predictor of achieving ≥ 70% total RDI, whereas postoperative complications, particularly readmission and CR‐POPF, were linked to a lower likelihood of reaching higher RDI.

The RDI threshold identified in this study is consistent with those reported in prior research. For example, a study by Wu et al. found that achieving ≥ 56% RDI significantly improved OS and that neoadjuvant chemotherapy increased the likelihood of reaching this threshold [15]. Yabusaki et al. found a cutoff of 80% RDI to be associated with better OS [14], while another study by Lee et al. demonstrated that a cumulative RDI ≥ 71.2% was optimal for radiological response [16]. A recent randomized trial found that patients with ≥ 85% chemotherapy dose density (DD) had higher OS compared to those with < 85% DD, though this study did not address the time element of chemotherapy delivery [17]. Additionally, a meta‐analysis showed that receiving < 85% RDI of FOLFOX‐, FOLFIRI‐, or FOLFIRINOX‐based regimens in pancreatic cancer patients was associated with a HR of 1.39 compared to patients with ≥ 85% [18].

The optimal treatment sequence for PDAC remains an area of active investigation [19, 20]. Our findings suggest that achieving ≥ 44% RDI in the neoadjuvant setting is associated with a higher rate of preoperative CA 19‐9 normalization and a greater likelihood of completing ≥ 70% total RDI. Consistent with these findings, multiple studies have demonstrated the benefits of neoadjuvant chemotherapy. Michelakos and colleagues found that compared to upfront resection, neoadjuvant mFOLFIRINOX significantly prolonged both DFS and OS in patients with borderline resectable or locally advanced PDAC [21]. Additionally, the PREOPANC phase III randomized trial found preoperative chemoradiotherapy to significantly improve DFS and reduce local recurrence, pathologic lymph node involvement, perineural invasion, and venous invasion. Among resected patients who received adjuvant chemotherapy, those receiving preoperative chemoradiotherapy had longer survival compared to those who did not [22].

With the growing evidence supporting the benefits of neoadjuvant therapy, an important question arises: For patients who undergo neoadjuvant treatment, do they still require adjuvant therapy, and which patients would benefit most from it? Several studies have shown that adjuvant chemotherapy is associated with higher OS and DFS in patients who have undergone neoadjuvant therapy and surgical resection, particularly for patients with elevated CA 19‐9 after neoadjuvant therapy [23, 24]. The benefit of adjuvant therapy appears to be influenced by nodal status and perineural invasion. One study found that the survival advantage associated with adjuvant therapy was limited to patients with pathology‐confirmed node‐positive disease following neoadjuvant therapy and resection [25], while another found pronounced survival benefit in patients with perineural invasion [26]. Furthermore, patients who achieve a stronger pathological response to neoadjuvant therapy (Evans IIa, IIb, or III) have been shown to derive greater benefit from adjuvant chemotherapy [27].

Our study found several predictors of achieving high RDI, with neoadjuvant therapy being associated with a higher odds and postoperative readmission and CR‐POPF associated with lower odds of high RDI. A study with 317 resected PDAC patients revealed that among the 25.2% of patients who did not receive adjuvant therapy, the most common reason was postoperative complications (38.8%). It also found being older and developing POPF to be independent predictors of not receiving adjuvant therapy [28]. Similar findings have been reported in other studies, documenting an over twofold increase in not receiving adjuvant therapy in patients experiencing severe postoperative complications [29, 30].

Importantly, this study suggests a synergistic effect between chemotherapy intensity and tumor biology, as achieving both high RDI and CA 19‐9 normalization confers the greatest survival benefit. These patients achieved prolonged OS with median OS of 62.6 months, which is comparable to outcomes reported in the PRODIGE trial [31]. While elevated postoperative CA 19‐9 level has been established as a marker of poor prognosis [32, 33, 34], other studies have also explored the prognostic utility of post‐neoadjuvant CA 19‐9 levels. For example, one study showed that adjuvant therapy significantly improved survival in patients with ≤ 50% preoperative CA 19‐9 reduction, suggesting the role of neoadjuvant CA 19‐9 response in predicting the additional benefit of adjuvant therapy [35]. A systematic review showed that optimal CA19‐9 response (> 50% reduction) after neoadjuvant treatment was significantly associated with improved prognosis, with no significant prognostic difference between optimal CA19‐9 response and normalization [36].

Additionally, in our cohort, all 13 patients with CA 19‐9 < 37 at diagnosis who received ≥ 70% RDI were alive at the time of analysis, suggesting that patients with normal CA 19‐9 may still benefit from high‐dose chemotherapy. Since CA 19‐9 cannot track disease progression in non‐secretors, data on the utility of intensive chemotherapy in this group remain limited. Our findings encourage further research on optimal treatment approaches for these patients, potentially incorporating developing strategies such as liquid biopsy to monitor response to treatment [37, 38].

This study has inherent limitations. As a single‐institution analysis, the RDI cutoffs identified in our cohort may not be directly applicable to other institutions or treatment settings. Additionally, exclusion due to missing dosage data could have introduced selection bias. Lastly, due to limited sample size, we were unable to separately analyze CA 19‐9 non‐producers (undetectable CA 19‐9, < 3 U/mL) from patients with normal levels (< 37 U/mL). Despite these limitations, this study provides a detailed analysis of the relationship between RDI, chemotherapy cycles, CA 19‐9 response, and survival outcomes. By providing a more precise measure of chemotherapy intensity, our findings underscore the clinical significance of RDI in perioperative management and highlight the need for future multicenter studies to validate and refine RDI‐based treatment strategies for PDAC.

## 5 Conclusion

Receiving a higher perioperative RDI in patients with resected PDAC treated with mFOLFIRINOX was associated with increased CA 19‐9 normalization and improved OS. Our results encourage a shift toward the use of RDI as a more precise metric for optimizing treatment and survival outcomes.

## Ethics Statement

This study was approved by the University of Pittsburgh Institutional Review Board (STUDY19060135).

## Conflicts of Interest

The authors declare no conflicts of interest.

## Synopsis

A relative dose intensity (RDI) of ≥ 70% was associated with better survival in resected PDAC patients treated with FOLFIRINOX. The greatest survival benefit was observed when both ≥ 70% RDI and CA 19‐9 normalization at the end of therapy is achieved.

## Supporting information


**Supplementary Figure S1:** Calculation of single‐agent relative dose intensity.
**Supplementary Figure S2:** Restricted cubic spline plot of FOLFIRINOX relative dose intensity and relative mortality hazard.
**Supplementary Figure S3:** Relationship between FOLFIRINOX RDI and cycle number.

## Data Availability

The data that support the findings of this study are available on request from the corresponding author. The data are not publicly available due to privacy or ethical restrictions.
