# Supplementary material for: Optimizing Perioperative Management of Pancreatic Ductal Adenocarcinoma: Insights Into Modified FOLFIRINOX Relative Dose Intensity and CA 19‐9 Dynamics
Source: J Surg Oncol. 2025 Jul 31;132(4):695–705. doi: 10.1002/jso.70057 (PMC12455553; doi:10.1002/jso.70057)
Supplement: Supplementary file 1 — Supplementary Figure S1: Calculation of single‐agent relative dose intensity. Supplementary Figure S2: Restricted cubic spline plot of FOLFIRINOX relative dose intensity and relative mortality hazard. Supplementary Figure S3: Relationship between FOLFIRINOX RDI and cycle number. [file JSO-132-695-s001.docx]

**Supplementary Figure 1.** Calculation of single-agent relative dose intensity

**Supplementary Figure 2.** Restricted cubic spline plot of FOLFIRINOX relative dose intensity and relative mortality hazard

**Supplementary Figure 3.** Relationship between FOLFIRINOX RDI and cycle number
